# Supplementary material for: Pan-Cancer Analysis of Homologous Recombination Deficiency in Cell Lines
Source: Cancer Res Commun. 2024 Dec 6;4(12):3084–98. doi: 10.1158/2767-9764.CRC-24-0316 (PMC11621922; doi:10.1158/2767-9764.CRC-24-0316)
Supplement: Figure S1 — CHORD MSI predictions [file crc-24-0316_figure_s1_suppsf1.pdf]

Supplementary Figure S1

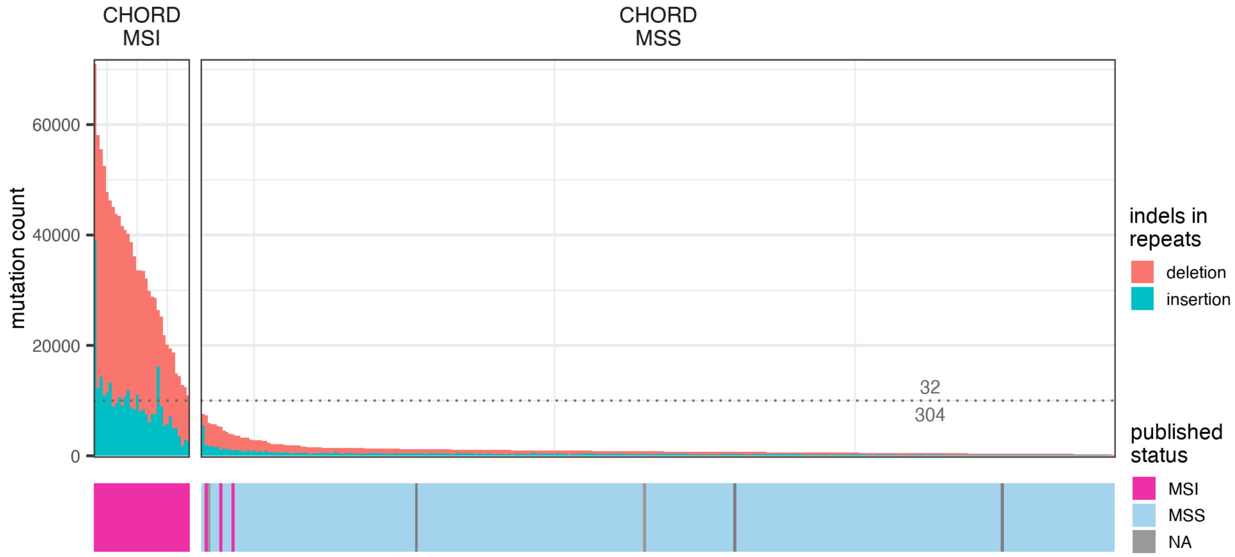

Supplementary Figure S1. CHORD MSI predictions. Top plot: Cell lines ranked by the number of indels present in repetitive regions. Cell lines with > 10,000 indels in repetitive regions were classified by CHORD as showing microsatellite instability (CHORD MSI) and were therefore ineligible for HRD assessment by CHORD. The other 304 cell lines were classified by CHORD as showing microsatellite stability (CHORD MSS). Bottom plot: MSI and MSS calls determined in a previous study (Chan et al., 2019). NA, MSI status not available.
