# Supplementary material for: Pan-Cancer Analysis of Homologous Recombination Deficiency in Cell Lines
Source: Cancer Res Commun. 2024 Dec 6;4(12):3084–98. doi: 10.1158/2767-9764.CRC-24-0316 (PMC11621922; doi:10.1158/2767-9764.CRC-24-0316)
Supplement: Figure S8 — Additional analyses related to associations of HRD predictions with drug sensitivities [file crc-24-0316_figure_s8_suppsf8.pdf]

Supplementary Figure S8

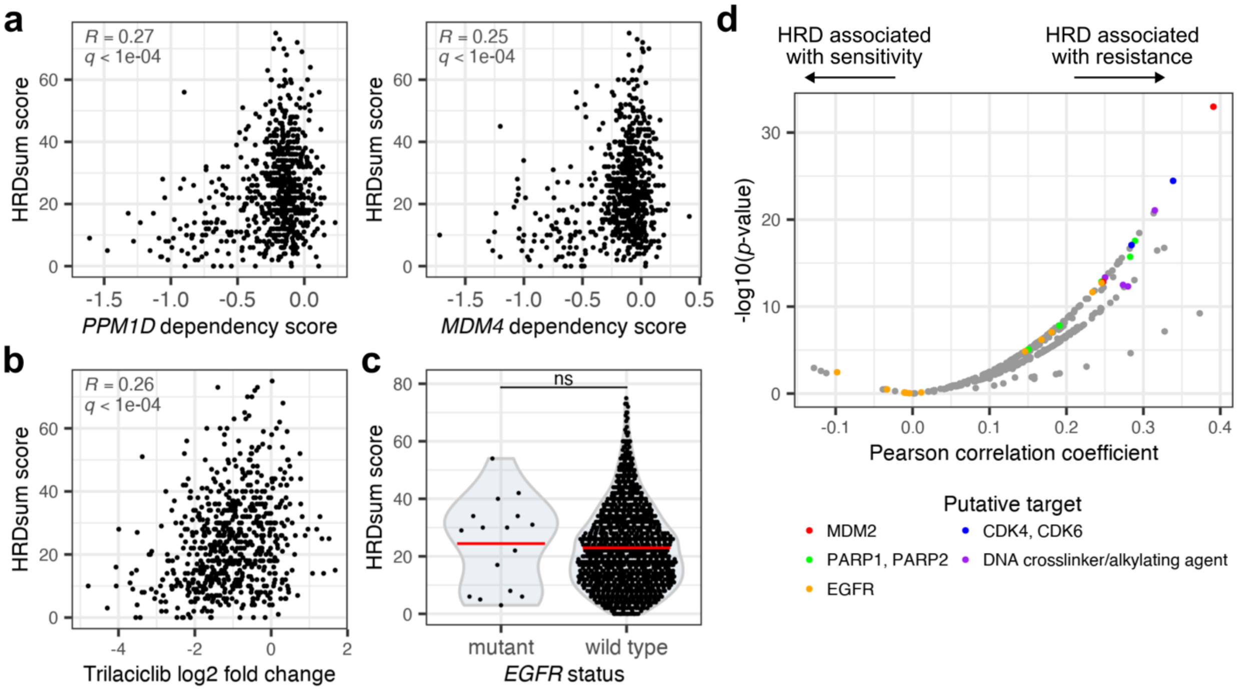

Supplementary Figure S8. Additional analyses related to associations of HRD predictions with drug sensitivities. a) Correlations between HRDsum scores and either *PPM1D* or *MDM4* dependency scores (DepMap 22Q4). Pearson correlation coefficients (*R*) and Benjamini-Hochberg-adjusted *p*-values (*q*) are shown in gray. **b)** Correlation between HRDsum scores and sensitivity to the CDK4/6 inhibitor Trilaciclib (PRISM Repurposing 23Q2). Sensitivity is shown as the log2 fold change in viability (treatment versus DMSO). Pearson correlation coefficient (*R*) and Benjamini-Hochberg-adjusted *p*-value (*q*) are shown in gray. **c)** HRDsum scores in relation to *EGFR* genotype. Cell lines harboring an *EGFR* mutation annotated as “pathogenic”, ‘likely pathogenic”, and/or “drug response” in ClinVar were classified as “mutant”. Cell lines lacking detectable variants in *EGFR* were classified as “wild type”. Red bars represent the mean. ns, not significant (Mann-Whitney U test). **d)** Pearson correlation coefficients between HRDsum scores and the log10(IC_{50}) for drugs available from the GDSC2 dataset. Positive coefficients indicate an association between HRDsum scores and drug resistance, whereas negative coefficients indicate an association between HRDsum scores and drug sensitivity. Colors highlight different drug targets of interest.
