# Supplementary material for: Pan-Cancer Analysis of Homologous Recombination Deficiency in Cell Lines
Source: Cancer Res Commun. 2024 Dec 6;4(12):3084–98. doi: 10.1158/2767-9764.CRC-24-0316 (PMC11621922; doi:10.1158/2767-9764.CRC-24-0316)
Supplement: Figure S7 — HRD predictions in relation to PARP inhibitor and platinum-based treatment response [file crc-24-0316_figure_s7_suppsf7.pdf]

Supplementary Figure S7

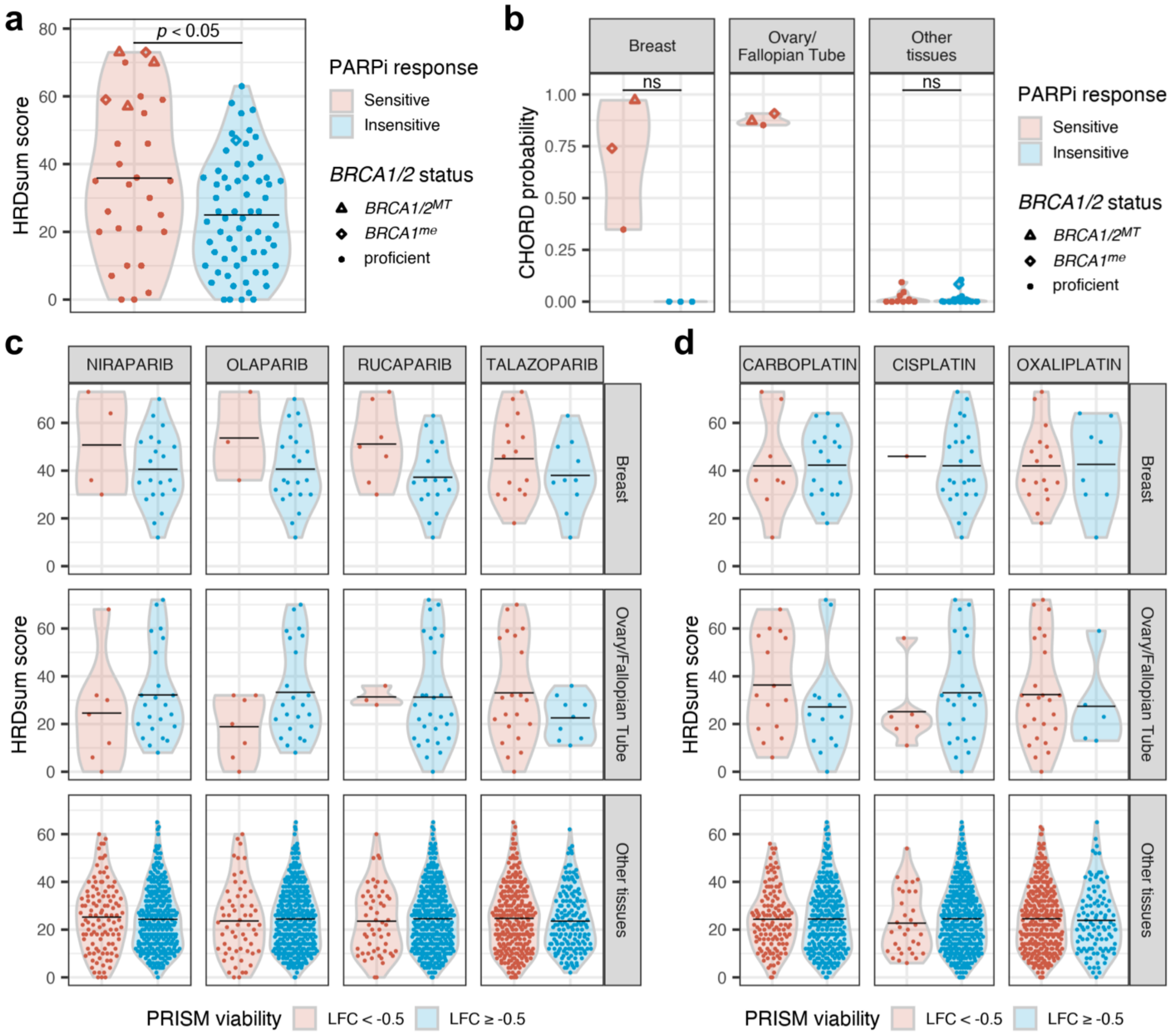

Supplementary Figure S7. HRD predictions in relation to PARP inhibitor and platinum-based treatment response. a) – b) HRD predictions in cell lines grouped by response to PARP inhibition, as measured by a clonogenic assay. Point shapes indicate biallelic loss of *BRCA1/2* (*BRCA1/2*^{MT}), likely epigenetic silencing of *BRCA1* (*BRCA1*^{me}), and likely BRCA1/2 proficiency (proficient). Black bars represent the mean. Mann-Whitney U test *p*-values are shown. ns, not significant. **c) – d)** HRDsum scores in cell lines grouped by response to various PARP inhibitors **(c)** and platinum-based chemotherapy drugs **(d)** using the PRISM Repurposing dataset (23Q2). Cell lines were grouped based on the log2 fold change in viability (treatment versus DMSO) using a cut-off of -0.5. Black bars represent the mean. None of the comparisons were significant (Mann-Whitney U test).
