# Supplementary material for: Pan-Cancer Analysis of Homologous Recombination Deficiency in Cell Lines
Source: Cancer Res Commun. 2024 Dec 6;4(12):3084–98. doi: 10.1158/2767-9764.CRC-24-0316 (PMC11621922; doi:10.1158/2767-9764.CRC-24-0316)
Supplement: Figure S6 — Additional associations of HRD predictions with genomic features and genetic dependencies [file crc-24-0316_figure_s6_suppsf6.pdf]

Supplementary Figure S6

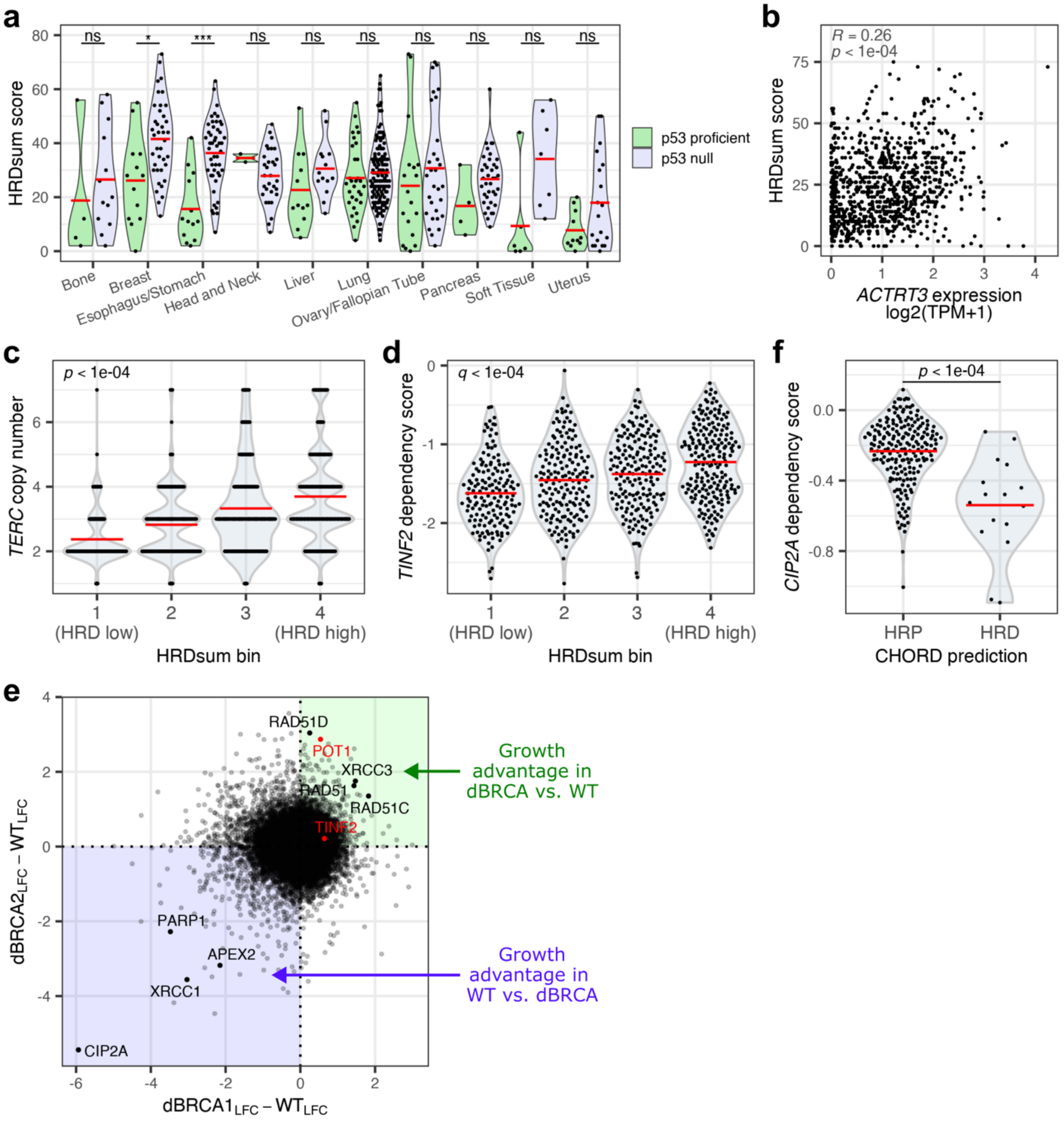

Supplementary Figure S6. Additional associations of HRD predictions with genomic features and genetic dependencies. a) HRDsum scores in p53-proficient cell lines versus p53-null cell lines, grouped by tissue type. Only tissue types with at least one HRDsum-high cell line are shown. Red bars represent the mean. ***, Benjamini-Hochberg-adjusted *p*-value < 0.001; *, Benjamini-Hochberg-adjusted *p*-value < 0.05; ns, not significant (Mann-Whitney U test). **b)** Correlation between HRDsum scores and *ACTRT3* expression (DepMap 22Q4). Pearson correlation coefficient (*R*) and *p*-value are shown in gray. **c)** *TERC* absolute copy number in cell lines binned by HRDsum score quartiles. Red bars represent the mean. Kruskal-Wallis test *p*-value is shown. **d)** *TINF2* dependency scores in cell lines binned by HRDsum score quartiles. Red bars represent the mean.

Q-value represents the Benjamini-Hochberg-adjusted *p*-value of a Kruskal-Wallis test. **e)** CRISPR knockout screen results for isogenic pairs of wild-type (WT) and either dBRCA1 (BRCA1-deficient) or dBRCA2 (BRCA2-deficient) cell lines. Axes show the difference in dBRCA and WT scores, which were calculated as the median-centered log fold change (LFC) in counts at the final timepoint vs. the initial timepoint. *POT1* and *TINF2* knockouts are highlighted in red. **f)** *CIP2A* dependency scores in CHORD-HRP cell lines versus CHORD-HRD cell lines. Red bars represent the mean. Mann-Whitney U test *p*-value is shown.
