# Supplementary material for: Pan-Cancer Analysis of Homologous Recombination Deficiency in Cell Lines
Source: Cancer Res Commun. 2024 Dec 6;4(12):3084–98. doi: 10.1158/2767-9764.CRC-24-0316 (PMC11621922; doi:10.1158/2767-9764.CRC-24-0316)
Supplement: Figure S5 — Alignment of HRD predictions to tumor types using Celligner [file crc-24-0316_figure_s5_suppsf5.pdf]

Supplementary Figure S5

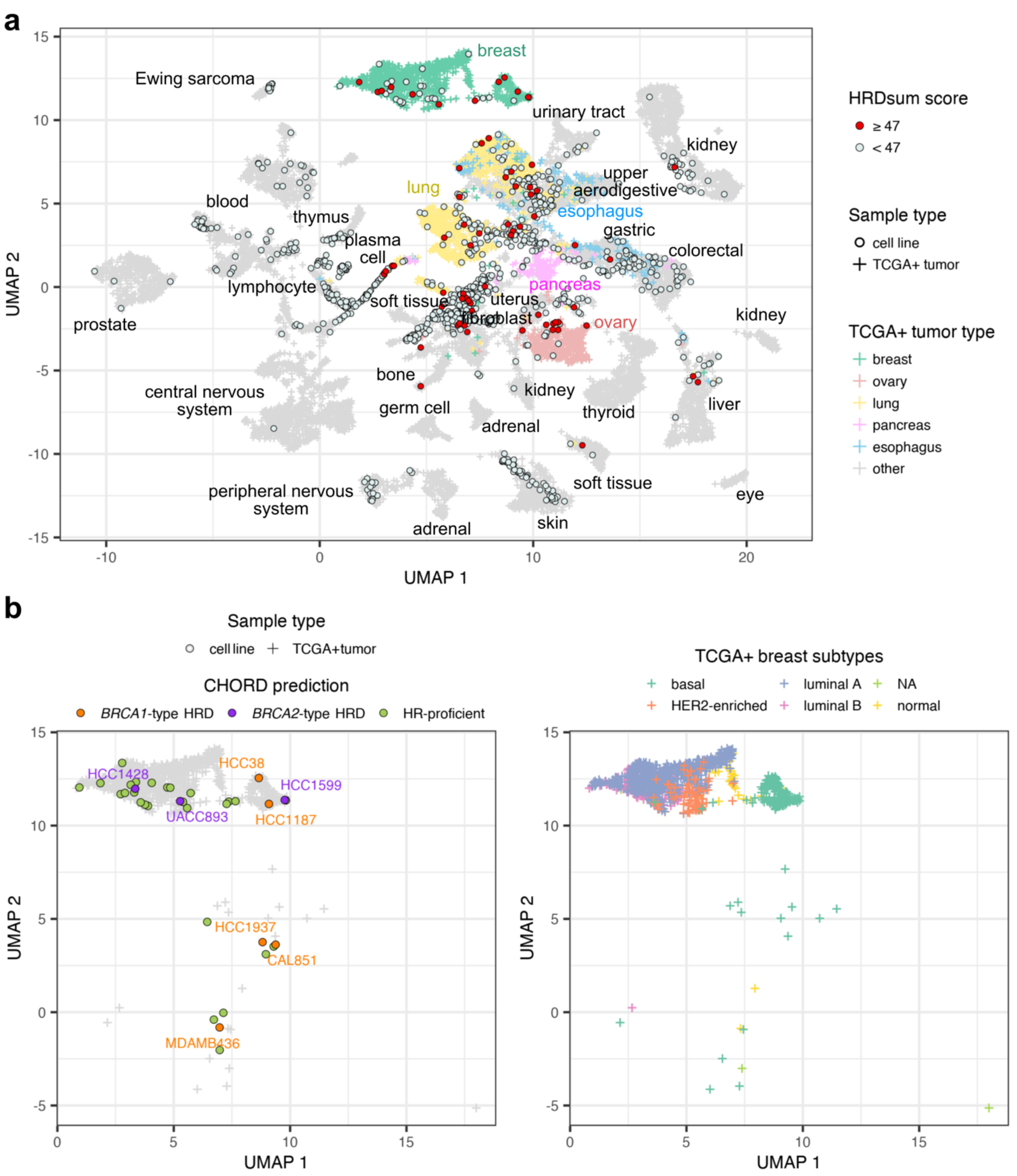

Supplementary Figure S5. Alignment of HRD predictions to tumor types using Celligner. a) Uniform Manifold Approximation and Projection (UMAP) plot of the Celligner dataset colored by HRDsum predictions. **b)** Left panel: UMAP plot of Celligner dataset for breast cancer samples colored by CHORD predictions. Right panel: UMAP plot of Celligner dataset for TCGA+ breast cancer samples colored by subtype.
