# Supplementary material for: Pan-Cancer Analysis of Homologous Recombination Deficiency in Cell Lines
Source: Cancer Res Commun. 2024 Dec 6;4(12):3084–98. doi: 10.1158/2767-9764.CRC-24-0316 (PMC11621922; doi:10.1158/2767-9764.CRC-24-0316)
Supplement: Figure S4 — Characterization of HRDsum scores in cell lines [file crc-24-0316_figure_s4_suppsf4.pdf]

Supplementary Figure S4

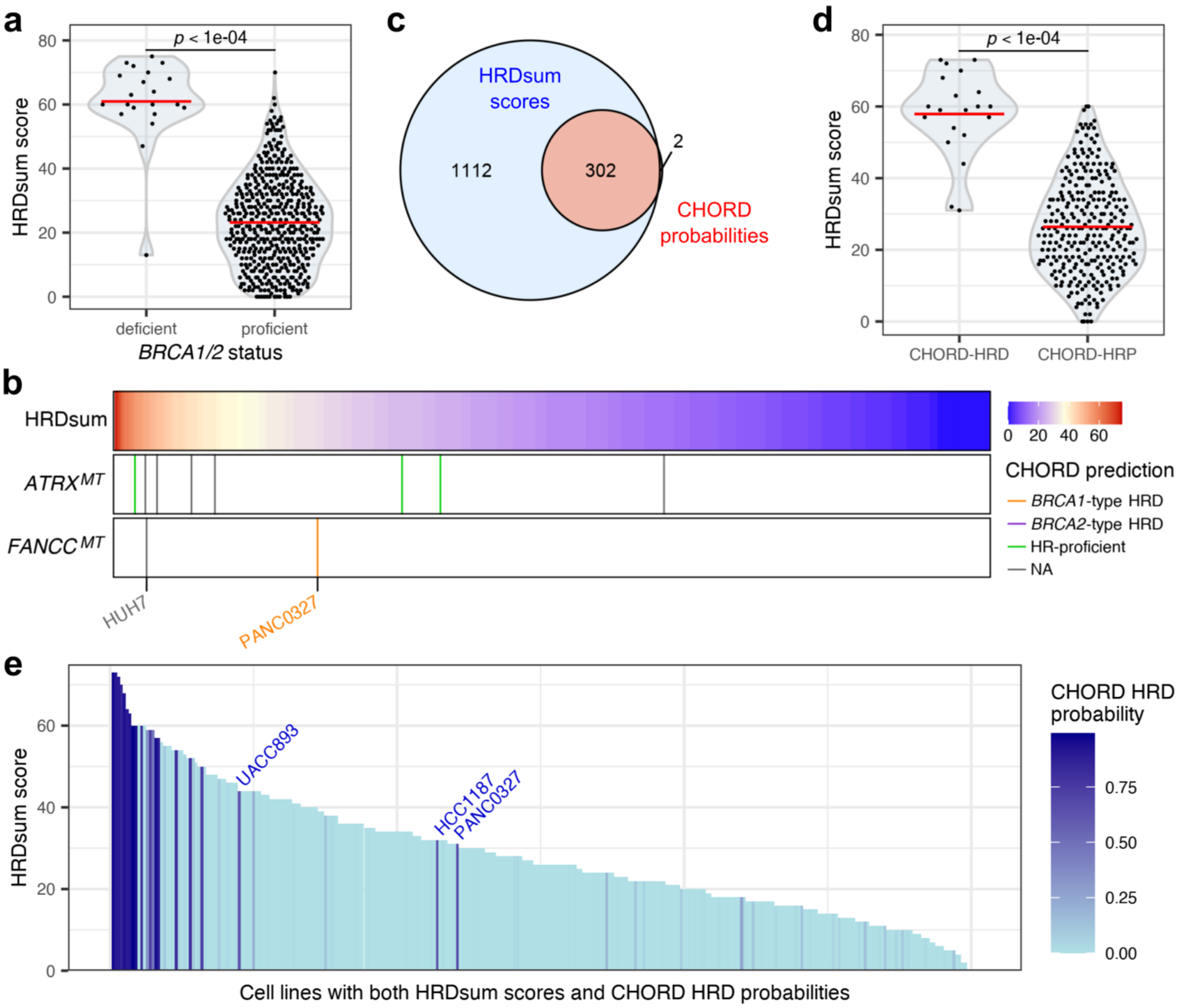

Supplementary Figure S4. Characterization of HRDsum scores in cell lines. a) HRDsum scores in cell lines grouped by BRCA1/2 functional status. BRCA1/2 deficiency was defined as either biallelic loss of *BRCA1/2* or likely epigenetic silencing of *BRCA1*. Red bars represent the mean. Mann-Whitney U test *p*-value is shown. **b)** *ATRX* and *FANCC* mutations in cell lines ranked by HRDsum score. Only biallelic loss-of-function mutations are shown. Mutant cell lines are colored by CHORD predictions, if available. NA, not available. **c)** Venn diagram showing the overlap in cell lines between the CHORD and HRDsum datasets. **d)** HRDsum scores in cell lines grouped by CHORD prediction. Red bars represent the mean. Mann-Whitney U test *p*-value is shown. **e)** Comparison of HRDsum scores (Y-axis) and CHORD HRD probabilities (color) for cell lines present in both datasets.
