# Supplementary material for: Pan-Cancer Analysis of Homologous Recombination Deficiency in Cell Lines
Source: Cancer Res Commun. 2024 Dec 6;4(12):3084–98. doi: 10.1158/2767-9764.CRC-24-0316 (PMC11621922; doi:10.1158/2767-9764.CRC-24-0316)
Supplement: Figure S3 — CHORD predictions relative to BRCA1 expression state [file crc-24-0316_figure_s3_suppsf3.pdf]

Supplementary Figure S3

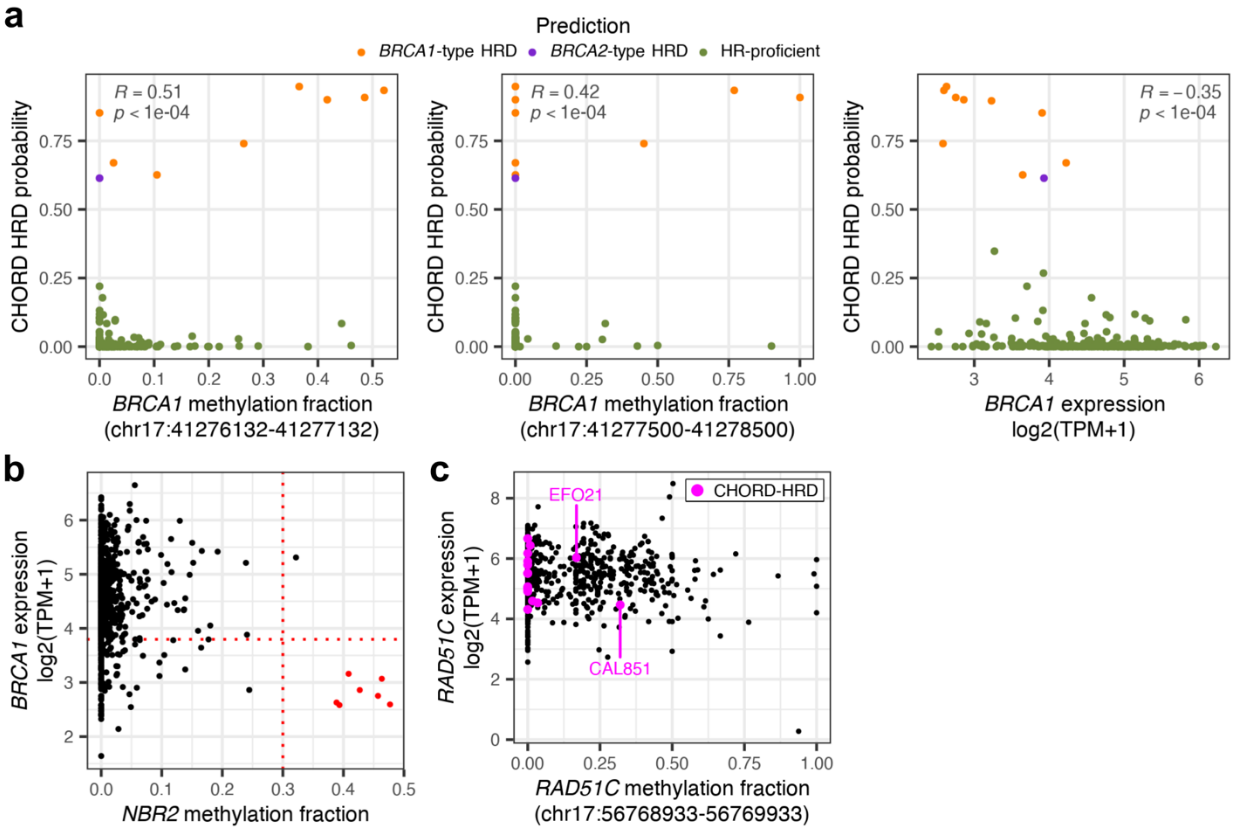

Supplementary Figure S3. CHORD predictions relative to *BRCA1* expression state. a) Correlations of CHORD HRD probabilities with *BRCA1* promoter methylation (left and middle panels) and *BRCA1* gene expression (right panel). Colors represent CHORD classifications. Pearson correlation coefficient (*R*) and Benjamini-Hochberg-adjusted *p*-values are shown in gray. **b)** Identification of cell lines showing evidence of *BRCA1* gene silencing. Likely cases of epigenetic silencing of *BRCA1* were determined by selecting cell lines with *NBR2* promoter methylation fraction > 0.3 and *BRCA1* expression levels below the lower quartile (red points). **c)** *RAD51C* expression and promoter methylation levels for cell lines analyzed by CHORD. CHORD-HRD cell lines are highlighted in magenta.
