# Supplementary material for: Pan-Cancer Analysis of Homologous Recombination Deficiency in Cell Lines
Source: Cancer Res Commun. 2024 Dec 6;4(12):3084–98. doi: 10.1158/2767-9764.CRC-24-0316 (PMC11621922; doi:10.1158/2767-9764.CRC-24-0316)
Supplement: Figure S2 — Comparison of CHORD and RAD51 foci assay results [file crc-24-0316_figure_s2_suppsf2.pdf]

Supplementary Figure S2

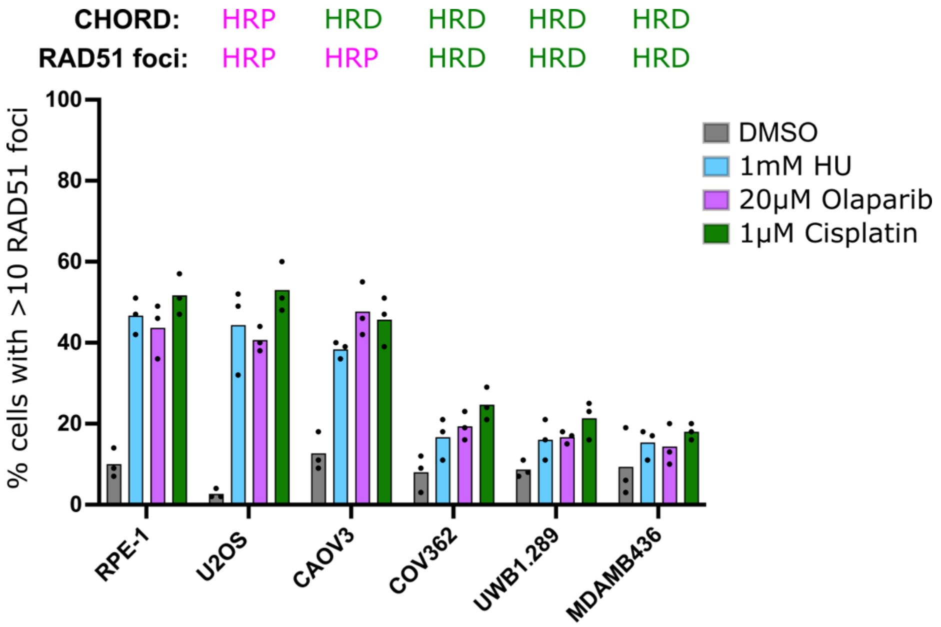

Supplementary Figure S2. Comparison of CHORD and RAD51 foci assay results. Quantification of cells that formed more than 10 RAD51 foci after treatment with the specified drug formulations. Three independent experiments were performed for each condition. Cell lines that showed RAD51 foci levels similar to those of RPE-1 cells (HR-proficient control) were called as HR-proficient (HRP), whereas cell lines that showed markedly lower levels of RAD51 foci compared to RPE-1 cells were called as HRD. A comparison of the results for the two assays is shown above the plot for each cell line. HU, hydroxyurea.
