# Supplementary material for: Pan-Cancer Analysis of Homologous Recombination Deficiency in Cell Lines
Source: Cancer Res Commun. 2024 Dec 6;4(12):3084–98. doi: 10.1158/2767-9764.CRC-24-0316 (PMC11621922; doi:10.1158/2767-9764.CRC-24-0316)
Supplement: Supplementary Table Captions [file crc-24-0316_supplementary_table_captions_suppstl.docx]

**SUPPLEMENTARY TABLE CAPTIONS**

**Supplementary Table S1. Mutation contexts for cell lines with WGS available.** Mutation contexts were extracted for SNVs, indels, and structural variants using the CHORD package in R.

**Supplementary Table S2. CHORD predictions for cell lines.** Includes the output of CHORD as well as cell line metadata. The “hr_status” and “hrd_type” columns were adjusted to an HRD cut-off of 0.4.

**Supplementary Table S3. Confusion matrix of CHORD HRD predictions in cell lines.** Labeled and predicted HRD classes using a CHORD cut-off of 0.4.

**Supplementary Table S4. DNA repair mutation enrichment analysis for CHORD predictions.** The “pct_hrd” and “pct_hrp” columns indicate the percent of CHORD-HRD and CHORD-HRP cell lines showing a deficiency in each gene, respectively. *P*-values and the Benjamini-Hochberg-adjusted *p*-values (*q*-values) are from a one-tailed Fisher’s Exact test. Only DNA repair genes with a deficiency in at least one CHORD-HRD cell line were analyzed.

**Supplementary Table S5. HRDsum scores for cell lines.** Columns beginning with “hrdsum” contain the raw HRDsum scores for the “Broad”, “Sanger (Sanger WES)”, and “Sanger (Broad WES)” datasets and the summary HRDsum scores derived from merging all three datasets.

**Supplementary Table S6. DNA repair mutation enrichment analysis for HRDsum scores.** The “pct_hrd” and “pct_hrp” columns indicate the percent of HRDsum-high and non-HRDsum-high cell lines showing a deficiency in each gene, respectively. *P*-values and the Benjamini-Hochberg-adjusted *p*-values (*q*-values) are from a one-tailed Fisher’s Exact test. Only DNA repair genes with a deficiency in at least one HRDsum-high cell line were analyzed.
